# Using Stakeholder Values to Promote Implementation of an Evidence-Based Mobile Health Intervention for Addiction Treatment in Primary Care Settings

**DOI:** 10.2196/13301

**Published:** 2019-06-07

**Authors:** Andrew Quanbeck

**Affiliations:** 1 Department of Family Medicine and Community Health University of Wisconsin-Madison Madison, WI United States

**Keywords:** implementation models, implementation strategies, mHealth, behavioral economics, game theory, decision-framing, primary care, stakeholder engagement

## Abstract

**Background:**

Most evidence-based practices (EBPs) do not find their way into clinical use, including evidence-based mobile health (mHealth) technologies. The literature offers implementers little practical guidance for successfully integrating mHealth into health care systems.

**Objective:**

The goal of this research was to describe a novel decision-framing model that gives implementers a method of eliciting the considerations of different stakeholder groups when they decide whether to implement an EBP.

**Methods:**

The decision-framing model can be generally applied to EBPs, but was applied in this case to an mHealth system (Seva) for patients with addiction. The model builds from key insights in behavioral economics and game theory. The model systematically identifies, using an inductive process, the perceived gains and losses of different stakeholder groups when they consider adopting a new intervention. The model was constructed retrospectively in a parent implementation research trial that introduced Seva to 268 patients in 3 US primary care clinics. Individual and group interviews were conducted to elicit stakeholder considerations from 6 clinic managers, 17 clinicians, and 6 patients who were involved in implementing Seva. Considerations were used to construct decision frames that trade off the perceived value of adopting Seva versus maintaining the status quo from each stakeholder group’s perspective. The face validity of the decision-framing model was assessed by soliciting feedback from the stakeholders whose input was used to build it.

**Results:**

Primary considerations related to implementing Seva were identified for each stakeholder group. Clinic managers perceived the greatest potential gain to be better care for patients and the greatest potential loss to be cost (ie, staff time, sustainability, and opportunity cost to implement Seva). All clinical staff considered time their foremost consideration—primarily in negative terms (eg, cognitive burden associated with learning a new system) but potentially in positive terms (eg, if Seva could automate functions done manually). Patients considered safety (anonymity, privacy, and coming from a trusted source) to be paramount. Though payers were not interviewed directly, clinic managers judged cost to be most important to payers—whether Seva could reduce total care costs or had reimbursement mechanisms available. This model will be tested prospectively in a forthcoming mHealth implementation trial for its ability to predict mHealth adoption. Overall, the results suggest that implementers proactively address the cost and burden of implementation and seek to promote long-term sustainability.

**Conclusions:**

This paper presents a model implementers may use to elicit stakeholders’ considerations when deciding to adopt a new technology, considerations that may then be used to adapt the intervention and tailor implementation, potentially increasing the likelihood of implementation success.

**Trial Registration:**

ClinicalTrials.gov NCT01963234; https://clinicaltrials.gov/ct2/show/NCT01963234 (Archived by WebCite at http://www.webcitation.org/78qXQJvVI)

## Introduction

### Context

The vast majority of practices shown to be effective by research remain unused in health care. It takes an estimated 17 years for an evidence-based practice (EBP) to be used in clinics, but only 14% of EBPs ever make it into use [1]. Mobile health (mHealth) technologies, in particular, hold great potential to transform health care. The evidence base for mHealth is limited but growing, with some technologies having been proven effective in randomized trials [2-5]. As of 2019, the degree to which mHealth technologies have been successfully implemented and integrated into the mainstream health care system in the United States remains limited.

The focus of this paper is a novel model for implementation that can generally be applied to EBPs. The model was developed through an exploratory analysis conducted in the context of an mHealth implementation research trial funded by the US National Institutes of Health (NIH) [6], making the results especially relevant to mHealth adoption. The trial involved 3 unaffiliated primary care clinics that enrolled 268 patients with substance use disorders to use a common mHealth system named “Seva,” a Hindi word that means “selfless caring.” As of 2019, the Seva implementation trial was among the most comprehensive mHealth implementation research trials reported in the US health care system, thus providing an instructive context for examining the emerging topic of mHealth implementation research. Little is known about the values and expectations stakeholders have regarding mHealth implementation [7]; this information needs to be brought forth and examined. Previous implementation research has focused on implementation frameworks and strategies [8-10], including frameworks specifically related to mHealth [11,12] and frameworks related to the definition and use of specific implementation strategies [10]. Substantial work has also been done to create tools to assess organizational readiness for change [13]. The decision-framing model contributes something new: a systematic approach that addresses the interactions between an mHealth intervention and the specific health system leaders, staff, and patients being asked to implement it.

### Theoretical Foundations

Fundamentally, implementing an EBP is a social process [14] involving human beings making decisions in the real world. Two areas of research provide important insights about the process of implementation but are rarely cited in implementation science: behavioral economics, which includes the concept of cognitive biases, and game theory. Both lines of research help explain how decision making works in the real world and why implementing an EBP is so challenging.

People frame their decisions on the basis of their own unique perspectives. Tversky and Kahneman’s paper, “The Framing of Decisions and the Psychology of Choice,” [15] defined a decision frame as “the decision maker’s conception of the acts, outcomes, and contingencies associated with a particular choice. The frame that a decision maker adopts is controlled partly by the formulation of the problem and partly by the norms, habits, and personal characteristics of the decision maker.” This paper lays out a decision-framing model for the implementation of an EBP—an mHealth intervention—used in primary care.

**Figure 1:**
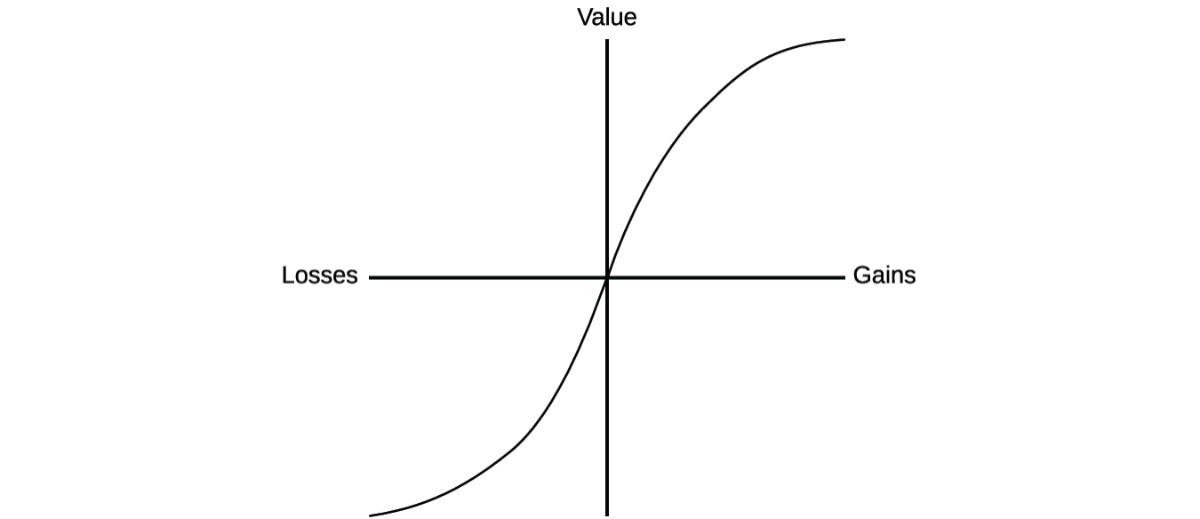
Schematic representation of decision framing in terms of gains and losses (adapted from Tversky and Kahnamen [15]).

Decision making involves not just a person’s perception of the acts, outcomes, and contingencies related to a specific choice, but also a subjective evaluation that determines the perceived value associated with a given choice. The perceived value of a particular choice depends critically on each decision maker’s unique perspective as a stakeholder in a health care system. In this context, *value* has a specialized meaning: It is the gains a person perceives in making a particular choice minus the losses the person perceives. Figure 1, which is adapted from Tversky and Kahnamen [15], illustrates the concept of perceived value in terms of the trade-off between perceived gains and losses.

This simple equation (wherein value equals perceived gains minus perceived losses) becomes complicated in light of Tversky and Kahneman’s pioneering work showing that the everyday choices people make are typically not governed by rationality, as had been long assumed in classical economic theory [15]. Instead, the perception of a decision—the way a choice is framed—can influence the option a decision maker selects. For example, people are generally risk seeking when the consequences of a choice are framed in negative terms and risk averse when consequences are framed positively—people will make a risky choice to avoid losing money but lock in a choice that involves a monetary gain. Indeed, people’s preferences can be reversed by the way a choice is put to them, and often be predicted by known cognitive biases (or heuristics). For example, agents of change (often, researchers) who encourage clinicians to adopt an EBP are likely to value the EBP more highly than prospective adopters do—sometimes because the agent has developed the practice himself or herself. This exemplifies a common decision-making bias called the *endowment effect,* that is, the tendency of individuals to ascribe inflated value to things for which they feel a sense of ownership. Decision makers are also highly *reference dependent;* inertia must be overcome to change from the status quo reference point that health care stakeholders begin with when considering a change in their routines. *Loss aversion* refers to decision makers’ preference, in the face of uncertainty, for avoiding losses over acquiring gains. Research across several domains has shown that perceived gains must outweigh losses by a substantial margin for decision makers to favor changing from the status quo [16]. This implies that a change agent must convince a potential adopter that changing practice is going to be highly preferential when compared with maintaining the status quo.

These decision-making biases are hard to address even among people well versed in them [17]. They combine to exert a powerful conservative force favoring the status quo with respect to clinical practice for both individuals and their work organizations.

Clearly, individual choices favor the status quo. Compounding the issue, implementation involves many different individuals making choices—all of them with different decision-making considerations. Game theory provides a framework for organizing implementation as a series of decisions made by members of different health care stakeholder groups [18]. According to game theory, all games comprise 4 key elements: players, actions, payoffs, and information. The central idea of this research is that success or failure in implementation is the result of decisions made by a diverse set of health care stakeholders, all of whom bring different perspectives and values to the decision of whether to adopt a given intervention. In health care, different stakeholders—payers (such as insurance companies), clinic managers, clinicians, and patients—are constantly confronted with choices about whether to adopt EBPs. Implementing an EBP may be conceptualized as a dynamic decision-making process that requires serial cooperation from all of these stakeholders to be successful.

In EBP adoption, the players can be defined as the 4 stakeholder groups named above (payers, clinic managers, staff, and patients); the players’ actions are either to adopt or resist adopting the EBP; and each player’s payoff corresponds to the perceived net benefits of adopting or not adopting *from that player’s perspective*.

At each stage of implementation, different stakeholder groups make informal assessments of the value of adopting the EBP. Abstaining from decision making by failing to participate in the implementation process is common and tantamount to not adopting. If members of a stakeholder group do not perceive that they will benefit significantly by adopting, they may choose not to adopt and maintain the status quo instead. For example, if management promotes an EBP that staff members find onerous, staff will likely not adopt unless they are strongly compelled to adopt. The serial cooperation required for successful implementation will be broken at this level, and patients will not have access to the EBP because their access depends on the cooperation of clinical staff. Staff members are acting rationally in this example, because, by not adopting, they are selecting the option with greater value *from their perspective*.

The game theory conception of implementation helps further explain the dismal statistics on implementation success cited at the start of this paper [1]. Generally, the decision of any stakeholder group not to adopt an EBP is likely to prevent the adoption of the EBP in the organization. Admittedly, this conception of the implementation process does not fully account for the complexity of implementation, which is in fact less linear and straightforward than the conception suggests. Behavioral economics and game theory both suggest that implementation is a complex human enterprise because it is a social process. The process involves people with different perspectives making decisions about the same choice—managing the implementation process is both science and art, with *a priori* odds overwhelmingly tilted toward failure.

### Aims

This paper provides a systematic model that implementers and researchers may use to gather input from health care stakeholders whose cooperation is essential to the successful implementation of EBPs. The model was applied specifically to an mHealth implementation study, and it therefore offers insights specific to mHealth, in addition to a method for designing and operating an effective implementation strategy. Someone who wants to introduce and implement an EBP into a system would benefit from understanding the considerations—the gains and losses—that potential adopters perceive as they think about adopting new practices. These considerations can then be used to modify the intervention or the implementation strategy (or potentially both) to better align with the considerations expressed by potential adopters and improve the likelihood of implementation success. Multimedia Appendix 1 describes the steps to implement the model.

## Methods

### The Focal Evidence-Based Practice

The mHealth intervention analyzed is called Seva, an evidence-based mHealth intervention designed to help prevent relapse in people recovering from substance use disorders [19,20]. Seva offers patients a discussion board used anonymously (with code names) by patients in the study; interactive modules that teach self-regulation, problem solving, and other skills; and health tracking tools and tools for coping with challenging situations, such as cravings and high-risk situations (eg, relaxation exercises, strategies from cognitive behavioral therapy, and links to local 12-step meetings). Seva gives clinicians a Web portal with a Clinician Report containing longitudinal information generated by patients’ self-reported data about their substance use and well-being (eg, sleep, depression). Seva (under the name A-CHESS) was proven effective in a randomized trial of patients leaving residential treatment for alcohol use disorders [20]. It is currently being tested in other substance use treatment contexts.

### Ethics Approval and Consent to Participate

The study protocol was designated minimal risk and approved by the University of Wisconsin’s Health Sciences Institutional Review Board (protocol number: 2012-0937-CP019). The parent study is registered with ClinicalTrials.gov (NCT01963234).

### Setting and Participants

The parent study [6] introduced Seva in 3 Federally Qualified Health Centers, which are primary care clinics in the United States that offer both primary and behavioral health care services to patients regardless of their ability to pay. At each of the 3 clinics, staff and patient participants were recruited for this exploratory analysis as a convenience sample from the staff and patients who consented to participate in the parent implementation study. Individual and group interviews were conducted with these stakeholders to elicit values related to the adoption of Seva and other EBPs. These interviews occurred during clinic visits from February 24 to 25, 2016; August 2, 2016; and September 21, 2016. These dates roughly corresponded to the transitional period between the stages of active implementation and maintenance in the parent study’s implementation plan. In total, 6 clinic managers, 17 clinical staff, and 6 patients were involved in the individual and group interviews. Table 1 shows characteristics of the patients and staff who participated in the interviews.

### Eliciting Stakeholder Considerations

**Figure 2:**
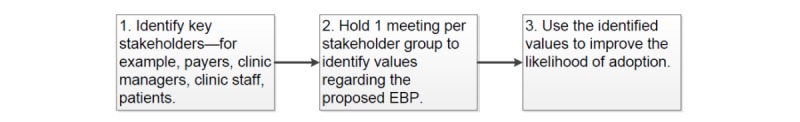
Decision-framing model. EBP: evidence-based practice.

The model used to frame decisions around EBP adoption is based on procedures for eliciting stakeholder considerations and defining a decision-analytic structure described by Edwards et al in their 2007 text, *Advances in Decision Analysis* [21]. The process is represented in broad terms by Figure 2. The type of decision analysis described here relies on a process of inductive reasoning in which the decision analyst constructs a model of the decision-making process by interviewing stakeholders. This model formulation is a first step; the result may then be tested prospectively in subsequent research and refined as necessary. The series of stakeholder interviews took place one-on-one and in group interviews with clinic managers, clinic staff, and patients. The decision analyst (AQ) explained the premise of decision framing: that different groups of stakeholders have different considerations and contexts depending on their role in the health care system and that these considerations bear on their decisions about adopting EBPs. The objective of the interviews was to elicit the considerations that could be translated into values and serve as the foundation of a decision-framing model for mHealth implementation from the perspective of different stakeholder groups. The interviews were semistructured and exploratory. A series of planned questions were asked to promote the discussion of key issues around implementation from each stakeholder perspective, followed up with probing questions to understand the ideas that participants expressed—the potential gains (pros or advantages) and losses (cons or disadvantages) derived from implementing and using Seva.

### Clinic Manager Interviews

Clinic managers at each implementation site were interviewed one-on-one by the decision analyst. Clinic managers continually make decisions about whether to undertake new projects, such as implementing Seva. Such decision making often occurs in the context of formal meetings intended to establish consensus around organizational goals (eg, monthly board meetings). An initial question posed during one-on-one interviews with managers from each site was, “What factors do you consider in deciding whether to introduce a new EBP like Seva to the staff and patients in your organization?” This initial inquiry was followed with specific questions about the factors the manager named, as well as questions that arose in the context of the discussion. Follow-up questions included the following: “At the organizational level, is there a process for deciding what new practices to implement? What barriers did you face in introducing Seva to your clinic? What would make it easier for you to implement Seva?”

### Clinic Staff Interviews

Teams of staff members who participated in the implementation of Seva were interviewed in a group setting at each of the 3 implementation sites. When it comes to adopting a new EBP, clinic staff members can usually choose to adopt the new practice (such as Seva) or maintain the status quo.

During group interviews with staff members, the decision analyst asked participants to reflect on the following question: “What do you think about when you are asked to do something new—a new procedure, a new technology, or some new evidence-based practice?” The decision analyst then gave staff members time to generate ideas individually. These ideas were then shared within the group in a round-robin fashion. After eliciting key considerations with respect to adopting new practices, the decision analyst used open-ended questions to expand on concepts presented by participants. Follow-up questions included the following: “In your different roles, how are you judged to be successful? Are metrics used (eg, number of patients seen, patient surveys, and other things)?”

**Table 1:** Baseline characteristics of participating clinics, clinic staff, and patients.

Characteristics			Site 1 (Madison, Wisconsin): Primary care and mental health	Site 2 (Missoula, Montana): Primary care, mental health, and addiction treatment	Site 3 (Bronx, New York): Primary care and mental health
**Clinic staff and roles, n**					
	Participants		5	9	9
	Manager		1	2	3
	Physician		1	2	1
	PhD psychologist		1	0	0
	Therapist, counselor, or social worker		0	2	3
	Care manager		0	2	1
	Medical assistant		2	0	0
	Clinic data manager		0	1	0
	Other		0	0	1
**Patients**					
	Participants, n		0	3	3
	Age (years), range		—^a^	43-56	40-63
	Gender (female), n		—	2	1
	**Highest education achieved, n**				
		Some high school	—	0	3
		Some college	—	2	0
		Associate’s degree	—	1	0
	**Drug of choice, n**				
		Alcohol	—	2	0
		Cocaine	—	0	1
		Marijuana	—	0	2
		Multiple drugs	—	0	1
	Ethnicity, Hispanic/Latinx, n		—	0	1
	**Race, n**				
		White	—	3	1
		African American/black	—	0	2

^a^Not applicable.

### Patient Interviews

Patients were interviewed in a group setting at both the Missoula and Bronx sites. (Owing to staggered implementation timing and turnover of a key staff member, patients at the first implementation clinic could not be reached for follow-up interviews). During these group interviews, patients were asked to reflect upon the considerations they had about adopting Seva and upon how Seva complemented other addiction treatment options, such as outpatient addiction treatment services offered by the clinic and traditional Alcoholics Anonymous/Narcotics Anonymous meetings. An initial question posed to patients was, “Assume you have a friend struggling with drug or alcohol problems who wants to know if you would recommend Seva to him or her. What would you say and why?” Each patient participant reflected on this question and shared responses, prompting group discussion. Follow-up questions posed by the decision analyst included the following: “What problems (if any) did you have using Seva? To what extent was cost a barrier to your using Seva?”

### Developing the Decision-Framing Model and Assessment of Face Validity

Input derived from this series of stakeholder interviews was used to establish the primary values (ie, trade-offs between perceived gains and losses) that governed stakeholders’ decisions about the implementation of Seva. Considerations gathered through the interviews were systematically reviewed by the decision analyst and another researcher, and they were compiled into a decision-framing model that expressed the considerations as perceived gains and losses from the perspective of each stakeholder group.

The first-order approach to assessing the validity of any model focuses on face validity—that is, the degree to which the model concords with holistic judgments of validity by the stakeholders whose input was used to develop it [22]. After the initial decision-framing model was constructed, a draft version of the manuscript was emailed to 6 of the clinic managers and staff members from the 3 clinics whose feedback was central in development of the decision-framing model. (Owing to practical concerns, chiefly having to do with privacy, feedback from patients was not solicited.) The respondents were asked to independently rate the degree to which they thought the decision-framing model incorporated the most important values related to their implementation of Seva and whether it provided a reasonable representation of the implementation decision-making process. These 6 stakeholders, whose considerations informed the decision-framing model, were offered the opportunity to engage in follow-up phone and email correspondence to provide feedback on face validity.

## Results

### Results From the Parent Study

The subsequent results and discussion should be understood in the context of the parent implementation study [6], which showed that implementation and effectiveness outcomes were largely positive; management supported the use of Seva in all 3 clinics, 1 or more clinic champions emerged at each site to engage and support patients, and patients showed significant reductions in drinking and drug use. Adoption results were mixed; although patients adopted Seva with very high levels of use by normal mHealth standards, use of Seva did not penetrate primary care clinical processes beyond use by a handful of clinic champions at each site. Maintenance of the system was unsuccessful; each clinic’s use of Seva ended when no long-term payer emerged to sustain the system after NIH grant funding ended.

### Implementation Considerations Expressed by Stakeholder Groups

Table 2 summarizes the implementation considerations that emerged during individual and group interviews in the context of the choices that are available to each stakeholder group.

**Table 2:** Stakeholder implementation considerations.

Stakeholder group	Decision alternatives	Considerations: perceived gains and losses	Notes on implementation
Clinic managers	Support implementation of Seva versus allocate resources to competing projects	Gain: increased quality of patient care; Loss: additional clinical staff time required to implement and operate the intervention; Gain: advances organizational mission; Loss: uncertainty about sustainability potential of new intervention; Loss: opportunity cost of time for clinic champion to lead change efforts; Loss: lack of integration of new intervention into existing clinical workflows	Perceived gains were evident at outset. Clinics were compensated for staff time during grant period to offset costs. Management at all clinics supported introduction and use of Seva throughout the implementation period. Though management at 2 of 3 sites supported ongoing use of Seva, the challenges of transferring from grant funding to a long-term sustainable operational plan could not be successfully addressed, and system use ended at all 3 sites
Clinic staff	Adopt Seva or maintain status quo clinical practice for addiction	Loss: time required to learn and use a new system; Loss: disruption of current workflows, including integration with the electronic health record (EHR); Gain: improved quality of patient care; Loss: uncertainty about long-term sustainability; Gain: potential to automate clinical functions currently done manually	Seva was heavily used and valued by clinic champions, but penetration beyond clinic champions was limited. Failure to integrate Seva data into EHR made accessing Seva data infeasible for most clinicians
Patients	Use Seva (in addition to standard addiction treatment offered by the clinic) or continue with standard addiction treatment offered by the clinic or seek other treatment (eg, Alcoholics Anonymous)	Gain: access to a safe means of recovery support (anonymous and private, as well as coming from a trusted source); Gain: promotes access to resources and connections to similar others; Gain: promotes autonomy in recovery management (ie, voluntary use on patient’s own time); Loss: cost to operate (including smartphone and data plan, covered by grant during intervention but transferred to patients after 12 months)	Patient out-of-pocket costs for Seva were paid with National Institutes of Health grant funding. Patient use during the study was high; use fell to zero when costs shifted to patients after grant funding ended. Logistical challenges made it difficult to transfer payment arrangements for data plans from the research team to individuals

### Clinic Managers

In determining whether to adopt Seva, clinic managers considered the greatest potential gain (or *advantage* or *pro*) to be providing better care for patients and the greatest potential loss (or *disadvantage* or *con*) to be costs, which were expressed in such terms as staff time, sustainability, and opportunity cost required to implement the change. In 1 clinic, if a proposed EBP would be valuable to patients, it is assessed for its alignment with other values expressed in the clinic’s mission—for example, the EBP should help build relationships with the community or foster the integration of medical, behavioral, and dental health services. This clinic follows a carefully designed process for deciding which innovations to adopt, using weekly management meetings of 5 clinic staff members. These meetings follow *Robert’s Rules of Order*, a widely used parliamentary protocol for conducting meetings and reaching group decisions [23]. In reaching a decision, the group also actively solicits clinician feedback. Nevertheless, innovations that improve patient care and align with organizational mission face the constraint of cost, which is ultimately synonymous with sustainability.

According to 1 clinic leader who championed the Seva project, “If a program like Seva costs us money, on balance, it will be very hard for us to sustain. If it saves the organization money, it has a chance.” As the director of behavioral health put it at another clinic, “We don’t want to spend the resources to create something new that won’t last.” Many Federally Qualified Health Centers are accountable care organizations (or part of such organizations) that are responsible for the total cost of care for each patient. If an intervention like Seva helps patients maintain healthy and stable lives—and helps avoid the costly emergency room visits, detoxification stays, and hospitalizations often associated with addiction—the cost of implementing it may be worthwhile. If an innovation cannot be paid for by a grant or insurance reimbursement, it is, by definition, unsustainable, and an unsustainable innovation is not worth implementing. Managers also weigh costs in terms of staff time and integration with existing workflows, and small aspects of a proposed innovation can affect those costs. One management group uses a visual representation of clinic workflows to see how an intervention might fit because altering existing operations and workflow is expensive in labor and opportunity cost. As this manager put it, “How many hoops will staff have to jump through to do this?”

### Clinic Staff

At all 3 clinics, virtually every clinic staff member interviewed cited time requirements as the foremost consideration in deciding to adopt a new practice. An intervention that costs staff extra time (expressed in terms of learning and using a new method or methods) is perceived negatively; a proposed intervention that might save staff time is perceived positively. An addiction psychiatrist whose patients used Seva shed light on what clinicians are trying to accomplish in the limited time they have with patients:

I’ve got 20 minutes with each patient every 6 weeks, if they show up. If you’re my patient and you’re not getting better, I want to know what is going on in your life. Are you not taking the medications I’ve prescribed? Are you using alcohol or other drugs? Are you having trouble with your family? Are you not sleeping? What’s going on?

This clinician values learning about a patient’s problems as efficiently as possible—time is her most limited resource. An mHealth system like Seva has the potential to save clinicians time by continuously gathering and summarizing patient data so that they can quickly get an accurate picture of their patients’ lives. Clinicians also said (like managers) that sustainability was important in their decision making, but to clinic staff, sustainability meant, in part, integration with the electronic health record (EHR). This EHR consideration arose in the group interviews because Seva data could not be integrated into the EHR, which caused inefficiency and frustration among clinicians. Clearly, the EHR is central to clinicians because it structures and monitors clinical work. This sets a high bar for EBP implementation because making changes to the EHR can be so difficult. In the health system that 1 clinic is part of, incorporating data into the EHR from external systems is reportedly so onerous that integration takes place only when changes are legally mandated (eg, a change to the EHR was enacted only after the state legislature passed a law requiring physicians to check the state’s Prescription Drug Monitoring Program database before prescribing an opioid).

### Patients

Because patients were asked about their decision making in the context of the Seva project, their responses reflected their thoughts about Seva and addiction treatment more specifically than the responses of managers and clinic staff, whose comments reflected their experience with the adoption of EBPs generally. Patients cited safety as their foremost consideration with regard to Seva. In the context of recovering from a substance use disorder, the concept of safety includes anonymity and privacy, as well as feeling confident that the innovation came from a trusted source (ie, on the basis of a recommendation from the patient’s clinician). Patients chose to use Seva in part because they weighed it against specific alternatives—Alcoholics Anonymous or Narcotics Anonymous meetings or not receiving treatment—that felt less safe to them. Patients’ comments also suggested that a successful innovation—all 6 patients interviewed regarded Seva as a valuable intervention—must promote connection to others and access to recovery resources. In both the rural setting of Missoula, Montana, and the urban setting of the Bronx, New York, patients reported feeling isolated. A patient in the Bronx said it was hard to get to Alcoholics Anonymous meetings, especially those held at night, because of concerns for her physical safety and the temptation to use drugs. In Missoula, getting to meetings was challenging because of difficulties with transportation. Seva addressed the isolation of these patients by enabling them to connect with peers and get help 24/7. A participant from the Bronx described how Seva helped him when he was on the brink of relapse. He decided to use Seva to reach out to one of the group’s monitors (a member of the research team). “If I didn’t have that phone, I don’t know what would’ve happened,” he reported. He also said the following:

Reaching out through Seva was the only thing that I could’ve done at that moment. I just needed someone to talk to; to listen to what was going on with me; to give me a push in the right direction.

Finally, patients wanted to feel in control of their choices to use Seva rather than be coerced. “I like the fact that this is not something I’m forced to do,” said one woman from the Bronx. She also said the following:

I can do it [use Seva] when I want to. This is my option. If I don’t feel like listening, I won’t listen!

### Face Validity

The 6 clinic managers and staff members who were consulted to provide face validity offered no corrections to the results presented.

## Discussion

### Principal Findings

This research conceptualizes the problem of implementing EBPs in a new way, borrowing key ideas from behavioral economics and game theory and integrating them with stakeholder feedback. This new conceptualization applies to the implementation of EBPs generally, but in this case, it was applied to one of the largest mHealth implementation trials to date, thereby specifically producing insights about mHealth implementation. The presumption of implementation researchers is that it is valuable to adopt EBPs *per se*. In truth, stakeholders have considerations, biases, and points of view that often limit the perceived usefulness of an EBP. It is the role of the implementer to frame decisions regarding intervention adoption for different stakeholders in the context of their considerations and values. This means presenting alternatives (ie, constructing subjective value functions) and adjusting the framing of the decision to maximize the probability of a positive choice for each stakeholder. The decision-framing model produced clear considerations that stakeholder groups used to evaluate the implementation of Seva, suggesting that decision-framing may be used to elicit multiple stakeholder perspectives. These considerations may in turn be used to (1) adapt the intervention to be implemented, or (2) tailor the implementation strategy used to deliver the intervention in ways that address stakeholders’ most important considerations, or both. For example, the study revealed that finding a way to pay for Seva was essential to sustaining it after the study ended. If we had learned this before rather than after implementation, we could have tried to address this more robustly at the beginning of implementation. As an example of how stakeholder considerations can be incorporated into tailoring an implementation strategy, suppose clinical staff express concern about the additional time required to implement a change in practice. In response, an implementer could organize a meeting between management and clinicians to define the work required and then carve out dedicated time in a clinician’s schedule—say, an hour every Friday morning—to commit to the work of implementation. This time expenditure would likely be viewed as a worthwhile investment by management if doing so leads to reduced hospitalizations for patients.

Payers are key stakeholders in health care systems, but payers were not directly interviewed for this study. In interviews with clinic managers and through interactions with a payer at 1 site, a single payer consideration was perceived as dominant: cost. An EBP may be perceived positively if it reduces the total cost of care—as Seva showed the potential to do through its effects on hospitalizations and emergency room visits [6]—or it may be perceived negatively if it increases the cost of care or has a cost that cannot be reimbursed.

**Figure 3:**
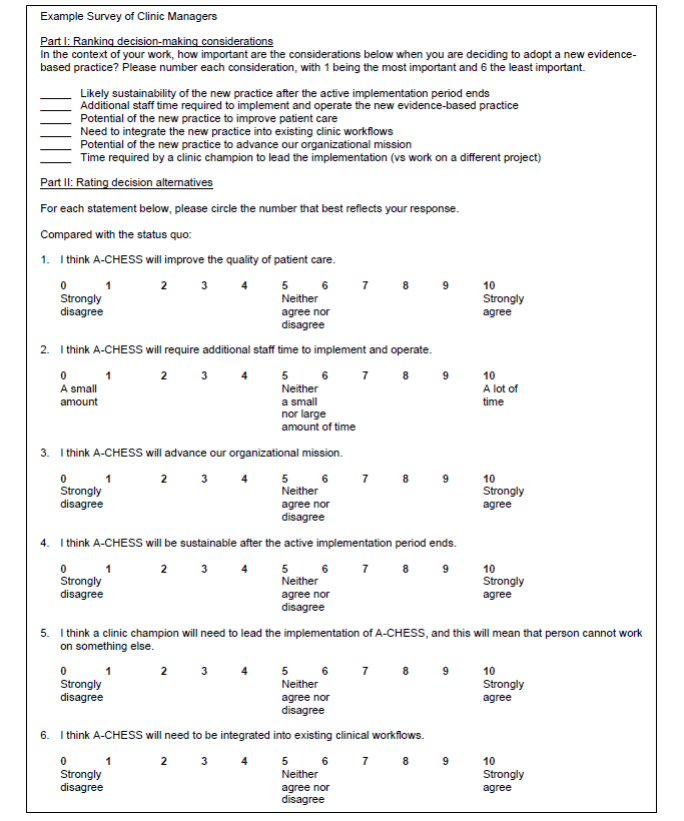
Illustration of prospective ranking and rating procedures.

The results of this inductively constructed model will now be tested prospectively, in a process using deductive reasoning, for its ability to predict the adoption of mHealth in a forthcoming implementation study funded by the US NIH (1R01DA04415901A1). In this test, the considerations reported here will be ranked (eg, clinic managers in the forthcoming trial will rank the 6 considerations reported by clinic managers in this paper), and then the decision alternatives will be rated (eg, clinic managers will rate how well the mHealth intervention addresses the considerations). See Figure 3 for an example section of the survey to be administered. The resulting rankings and ratings can then be weighted to determine how important they are to address, either by modifying the mHealth intervention or the strategy used to implement it. See the instructions for weighting considerations in Multimedia Appendix 1.

A nesting structure of values emerged through the interviews with stakeholders, both within each stakeholder group and between 1 stakeholder group and the next. For instance, management implied that for an intervention to be maximally appealing for adoption, it must first and foremost be valuable to patients, then palatable to staff, and then sustainable from a cost and reimbursement perspective. In a sense, management is implicitly incorporating the key values of patients, staff, and payers when deciding whether to approve implementation projects.

### Comparison With Previous Work

Although many potentially useful instruments and frameworks are available from the implementation research literature to aid in the implementation process [13], the decision-framing model (1) provides a systematic approach for assessing the perceived value of an intervention from multiple stakeholder perspectives, (2) is concise and pragmatic, and thus suitable for widespread application (in contrast to more comprehensive, research-oriented models and questionnaires), and (3) offers an intervention-specific model that accounts for the complex interactions among organizational leaders, staff, the intervention itself, and patients. (Models of implementation [8] suggest that all these factors are relevant to implementation success.) Multimedia Appendix 1 provides practical guidance on how to use the decision-framing model in implementation and implementation research.

The need for systematic tailoring of implementation strategies has been identified as essential in the implementation research literature [24,25], but determining exactly *how* to conduct effective tailoring is still an understudied area. Decision framing can provide an organizing structure to gather information at the start of implementation—for instance, during the Exploration and Adoption/preparation stages of Aarons’ framework [9]—as well as during active implementation. The process may yield useful information for tailoring implementation strategies on the basis of stakeholder values. External change agents, such as organizational coaches or facilitators, often lead implementation efforts. Research has shown that the effectiveness of facilitation can vary [26]. External change agents may benefit by using a systematic model, such as decision framing, because it provides a set of operating principles from which to orchestrate an implementation process that helps ensure that stakeholders have been involved and their values have been heard. Doing so could minimize the chance that implementation fails because of inconsistency in approaches to stakeholder engagement, a prospect that is virtually inevitable when left to the variability of human change agents.

### Limitations

For logistical reasons, the decision-framing model was constructed retrospectively, at roughly the end of the implementation period at each site. Stakeholders’ responses may have been different if the process had been undertaken before Seva was adopted. Prospective application of decision framing will take place in a forthcoming randomized trial, an NIH-funded implementation trial that was funded in 2018 (1R01DA04415901A1).

The face validity of decision framing was established in the context of a single study involving 1 type of health care setting (Federally Qualified Health Centers) and 1 EBP (an mHealth intervention for substance use disorders). Further research will be needed to validate the model and examine its usefulness with other interventions in other settings. The data reported also represent small samples, especially with only 6 patients interviewed, warranting caution about the generalizability of the findings.

Decision framing is a simple model that seeks to capture essential decision-making processes related to implementation research. More quantitatively robust decision-analytic techniques certainly exist (eg, multiattribute utility theory), but trade-offs are inevitable between pragmatism and research sophistication in selecting a model. Decision framing was selected in part because it is simple and intuitive enough for wider uptake.

Finally, decision modeling of any type invariably simplifies the complexity of any actual implementation process. Implementation does not always unfold in an orderly fashion, and assigning accurate weights to considerations can be difficult. For example, unreimbursed cost sealed the fate of Seva, despite patients’ positive perceived value and the efforts of leadership in 1 clinic to find funding, and it may be that unreimbursed cost commonly plays such a role in implementation. In addition, the implementation of some practices may not require cooperation from all stakeholder groups—for example, patients may choose to use certain EBPs (such as mHealth apps) without any support or involvement from clinic management or staff. Demand for innovations can bubble up from patients and staff; indeed, such origins may bode more favorably for successful implementation than the top-down approach to implementation that is common in the health care system.

### Conclusions

Though the decision-framing model is new to implementation research, the rationale for it is both simple and pragmatic: implementing an EBP is a fundamentally social process [8], and the inescapable biases associated with human decision making apply in implementation research just as they do in every other aspect of life. Decision-framing techniques have been exhaustively studied, validated, and applied in many fields, including psychology, business, and management. Innovation often lies in scanning many disciplines, making logical connections, and matching the most appropriate solutions available to the problem at hand. Newly applied to implementation research, decision-framing offers a potential tool for implementers to use in speeding the adoption mHealth interventions and other EBPs.

## Appendix

Multimedia Appendix 1 Decision-framing to incorporate stakeholder perspectives in implementation.

## Abbreviations

EBP: evidence-based practice
EHR: electronic health record
mHealth: mobile health
NIH: National Institutes of Health

## References

[1] Balas E, Boren S, Bemmel J, McCray AT (2000). Managing clinical knowledge for health care improvement. Yearbook of Medical Informatics 2000: Patient-Centered Systems.

[2] Johnston L, Zemanek J, Reeve MJ, Grills N (2018). The evidence for using mHealth technologies for diabetes management in low- and middle-income countries. J Hosp Manag Health Policy.

[3] Peiris D, Praveen D, Johnson C, Mogulluru K (2014). Use of mHealth systems and tools for non-communicable diseases in low- and middle-income countries: a systematic review. J Cardiovasc Transl Res.

[4] Sondaal SFV, Browne JL, Amoakoh-Coleman M, Borgstein A, Miltenburg AS, Verwijs M, Klipstein-Grobusch K (2016). Assessing the effect of mHealth interventions in improving maternal and neonatal care in low- and middle-income countries: a systematic review. PLoS One.

[5] Gustafson DH, McTavish FM, Chih M, Atwood AK, Johnson RA, Boyle MG, Levy MS, Driscoll H, Chisholm SM, Dillenburg L, Isham A, Shah D (2014). A smartphone application to support recovery from alcoholism: a randomized clinical trial. JAMA Psychiatry.

[6] Quanbeck A, Gustafson DH, Marsch LA, Chih M, Kornfield R, McTavish F, Johnson R, Brown RT, Mares M, Shah DV (2018). Implementing a mobile health system to integrate the treatment of addiction into primary care: a hybrid implementation effectiveness study. J Med Internet Res.

[7] Wallis L, Blessing P, Dalwai M, Shin SD (2017). Integrating mHealth at point of care in low- and middle-income settings: the system perspective. Glob Health Action.

[8] Damschroder LJ, Aron DC, Keith RE, Kirsh SR, Alexander JA, Lowery JC (2009). Fostering implementation of health services research findings into practice: a consolidated framework for advancing implementation science. Implement Sci.

[9] Aarons GA, Hurlburt M, Horwitz SM (2011). Advancing a conceptual model of evidence-based practice implementation in public service sectors. Adm Policy Ment Health.

[10] Powell BJ, Waltz TJ, Chinman MJ, Damschroder LJ, Smith JL, Matthieu MM, Proctor EK, Kirchner JE (2015). A refined compilation of implementation strategies: results from the Expert Recommendations for Implementing Change (ERIC) project. Implement Sci.

[11] Labrique AB, Vasudevan L, Kochi E, Fabricant R, Mehl G (2013). mHealth innovations as health system strengthening tools: 12 common applications and a visual framework. Glob Health Sci Pract.

[12] Wallis L, Hasselberg M, Barkman C, Bogoch I, Broomhead S, Dumont G, Groenewald J, Lundin J, Norell BJ, Nyasulu P, Olofsson M, Weinehall L, Laflamme L (2017). A roadmap for the implementation of mHealth innovations for image-based diagnostic support in clinical and public-health settings: a focus on front-line health workers and health-system organizations. Glob Health Action.

[13] Weiner BJ, Amick H, Lee SY (2008). Conceptualization and measurement of organizational readiness for change: a review of the literature in health services research and other fields. Med Care Res Rev.

[14] Rogers E (2010). Diffusion of Innovations.

[15] Tversky A, Kahneman D (1981). The framing of decisions and the psychology of choice. Science.

[16] Kahneman D, Lovallo D, Sibony O (2011). Before you make that big decision. Harv Bus Rev.

[17] Kahneman D (2013). Thinking, Fast and Slow.

[18] Myerson R (1991). Game Theory: Analysis of Conflict.

[19] McTavish FM, Chih M, Shah D, Gustafson DH (2012). How patients recovering from alcoholism use a smartphone intervention. J Dual Diagn.

[20] Gustafson DH, McTavish FM, Chih M, Atwood AK, Johnson RA, Boyle MG, Levy MS, Driscoll H, Chisholm SM, Dillenburg L, Isham A, Shah D (2014). A smartphone application to support recovery from alcoholism: a randomized clinical trial. JAMA Psychiatry.

[21] Edwards W, Miles R, von Winterfeldt D (2019). Advances in Decision Analysis: From Foundations to Applications.

[22] von Winterfeldt D, Edwards W (2007). Decision Analysis and Behavioral Research.

[23] Robert 3rd HM, Evans WJ, Honemann DH, Balch TJ, Seabold DE, Gerber S (2011). Robert's rules of order newly revised. 11th edition.

[24] Powell BJ, Beidas RS, Lewis CC, Aarons GA, McMillen JC, Proctor EK, Mandell DS (2017). Methods to improve the selection and tailoring of implementation strategies. J Behav Health Serv Res.

[25] Lewis CC, Scott K, Marriott BR (2018). A methodology for generating a tailored implementation blueprint: an exemplar from a youth residential setting. Implement Sci.

[26] Ritchie MJ, Kirchner Je, Parker Le, Curran Gm, Fortney Jc, Pitcock Ja, Bonner Lm, Kilbourne Am (2015). Evaluation of an implementation facilitation strategy for settings that experience significant implementation barriers. Implementation Sci.

