# Supplementary material for: Using Stakeholder Values to Promote Implementation of an Evidence-Based Mobile Health Intervention for Addiction Treatment in Primary Care Settings
Source: JMIR Mhealth Uhealth. 2019 Jun 7;7(6):e13301. doi: 10.2196/13301 (PMC6746086; doi:10.2196/13301)
Supplement: Multimedia Appendix 1 [file mhealth_v7i6e13301_app1.pdf]

Decision-framing to Incorporate Stakeholder Perspectives in Implementation

Understanding the values of healthcare stakeholders and increasing the likelihood of adopting an evidence-based practice

School of Medicine and Public Health
UNIVERSITY OF WISCONSIN-MADISON

DEPARTMENT OF FAMILY MEDICINE
University of Wisconsin
School of Medicine and Public Health

Health Innovation Program
Integrating healthcare research and practice

Contents

Use of the toolkit 3
Who should use this toolkit? 3
What does the toolkit contain? 3
Development of this toolkit 3
Background 4
Decision-making biases 5
Endowment effect 5
Loss aversion 5
Framing effects 5
An approach to solving the problem of implementation 7
1. Identify the key stakeholders 7
2. Hold group meetings of stakholder to identify the considerations they have, both positive and negative, about the change you propose 7
3. Weigh stakeholders' considerations to arrive at the value they attach to the change 8
4. Use the values to identify actions to improve the likelihood of adoption 8

Use of the toolkit

Who should use this toolkit?

This approach is intended for healthcare researchers, facilitators, and others who want to introduce an improvement in primary care clinics.

What does the toolkit contain?

This toolkit contains information on how people make decisions, and how common decision-making biases can affect the success of implementation projects.

It also includes steps that can be taken to convene stakeholders, identify their values, and use the values to take actions that improve the likelihood of adoption.

Development of this toolkit

The Decision-framing to Incorporate Stakeholder Perspectives in Implementation Toolkit was developed by researchers and clinicians (Principal Investigator: Andrew Quanbeck) at the University of Wisconsin-Madison School of Medicine & Public Health – Department of Family Medicine and Community Health.

The National Institute on Drug Abuse (NIDA) is the primary funder of the study (K01-DA039336-01). The funder had no role in study design, the collection or interpretation of data, or the publication of results. Additional funding was provided by NIDA grants R34-DA036720-01 and R01-DA030431-01.

Please contact Dr. Quanbeck with any questions regarding this approach or suggestions for improving it: arquanbe@wisc.edu

This toolkit, Decision-framing to Incorporate Stakeholder Perspectives in Implementation, is available at https://www.hipxchange.org/ImplementationDecisionMaking and licensed under a Creative Commons Attribution-NonCommercial-ShareAlike 4.0 International License.

The toolkit exists for the benefit of the health care community. These materials are available free of charge and can be used without permission; however, we ask that you register with HIPxChange before using the toolkit so that we may provide information on usage to our funders. It is acceptable to link to this Web site without express permission. If you decide to use these materials, we ask that you please use the citation below.

Citation: Andrew Quanbeck. Decision-framing to Incorporate Stakeholder Perspectives in Implementation toolkit. University of Wisconsin – Madison Department of Family Medicine & Community Health. Madison, WI; 2019. Available at: https://www.hipxchange.org/ImplementationDecisionMaking

Background

It takes an average of 17 years for an evidence-based practice (EBP) to be adopted into clinical practice—and most EBPs are never widely adopted. The decision-framing approach described below makes it easy to identify the values that different stakeholders have about implementing a possible change, potentially speeding the adoption and improving the effectiveness of EBPs. Stakeholders include, for example, payers, clinic managers, clinic staff, and patients. Once the different groups' values are known, the potential EBP and/or aspects of the clinic workflow and setting can be modified to raise the likelihood that the EBP will be adopted.

Implementing a new practice in an organization can be viewed as a process involving a group of healthcare stakeholders who all have to **cooperate** in a conscious way.

Implementation is a dynamic process that involves real people making decisions in the real world. At each level--from payers, to management, to staff, to patients--healthcare stakeholders are making decisions about whether to adopt the change you're trying to implement. If the people at one level decide not to adopt a proposed new practice, the practice probably will not be adopted. For example, if managers promote a practice that staff find onerous, staff are likely not to adopt it. Abstaining from decision-making by not participating in implementation is tantamount to not adopting. All the relevant decision makers need to be participating and ultimately to agree or the process is over and the implementer can go home. This happens nearly all the time. Remember, 17 years?

Decision-making biases

Daniel Kahneman, in his book *Thinking Fast and Slow,* talks about two types of decision making, System 1 and System 2. System 1 refers to the largely unconscious and reflexive mode of cognition where people spend the vast majority of their time. System 2 refers to the conscious, deliberate cognition that people draw upon when faced with making a choice.

To successfully implement a change, one of an implementer's first steps is engaging the groups of stakeholders in the implementation process so they know they are part of a dynamic process that requires decision making. This may seem trivial, but in reality, it is no small feat. Many projects fail because the people whose participation is essential to successful implementation don't know that they have choices to make.

Let's assume that you can at least get stakeholders to understand that a process is taking place. Even then, you want decision makers to make a specific choice--that is, to adopt the change. You still have a whole host of difficult decision-making biases to overcome. Let's consider a few examples.

Endowment effect

The endowment effect is the tendency of individuals to give inflated value to things they feel a sense of ownership for. A good example is a homeowner listing his or her home for sale. The house means a lot to someone who's lived in it for 20 years. Homeowners often think their house is worth more than a prospective buyer does. In fact, a house is worth exactly what a buyer is willing to pay for it--no more, no less.

In the same way, if you want to effect a change in healthcare practice, you've probably got a lot invested that change. Maybe you developed the change yourself. At the very least you probably have spent a lot of time learning about it. You've likely endowed the change with greater value than the stakeholders who must decide to adopt it. On the contrary, the decision maker is used to behaving in a certain way. It's the status quo. Changing behavior will require the decision maker to alter or even overturn the status quo.

Loss aversion

Loss aversion refers to the preference people have when they face a choice for avoiding losses over acquiring gains. Research shows that benefits, or advantages, must outweigh costs, or disadvantages, by about two to one to change the status quo. As an implementer, you have to convince someone that changing is going to be *twice as good* as not changing. It's a high bar.

Loss aversion thus acts as a powerful conservative force that favors the status quo for both individuals and the organizations they work in.

Framing effects

In implementation science, it is common to frame projects in terms of protocols that describe what staff members must do. But decision makers are naturally hesitant to adopt a change that is presented as a series of additional tasks. Why? Because most people believe that they're really busy at work and do not have time to take on anything additional. Whether this is someone's perception or a realistic assessment of workload doesn't really matter. Perception is the only reality that matters in this case.

It is best simply to recognize and accept this law of human nature. Very simply, you cannot ask someone to do more than they are currently doing. You have to convince the person that doing something different will be worthwhile. To that end, posing a question about whether a person has time to do something new produces no useful information because the answer is always "no." Instead, ask questions about what it is like to be in the other person's shoes. What about your job is rewarding? What is challenging? You can then use this understanding to frame the change you are advocating or revise the change so that it aligns with what the person said.

Other framing issues

Use positive language to describe the change you want to implement. Negative language doesn't work very well and may insult people. Would you want to get a report card that labeled you a "poor performer"? That said, people sometimes perceive a statement negatively no matter how it's phrased. Try things out--pilot test them--to understand how your efforts will be perceived.

Avoid slogans. An example: "Work smarter, not harder." If someone says this to you, what is the person suggesting about the way you currently work? Similarly, any variant of the slogan, "Help me help you" is patronizing.

An approach to solving the problem of implementation

This approach is intended for healthcare researchers, facilitators, and others who want to introduce an improvement in primary care clinics. It flows from the principles described above. The approach is meant to be a practical help for the person implementing a change. The approach fosters an understanding of the people and setting where the implementer wants to introduce a change. This understanding enables the implementer to adapt the change to improve the likelihood of implementation success. Instructions on how to solve the problem of implementation are listed below.

1. Identify the key stakeholders.

Who are the people affected by the change you propose? They are often payers, clinic managers, clinicians and other clinic staff, and patients, but other people could be affected as well. Think about all the people in an organization who would be affected by the change you want to make—all the people whose decision to adopt or not adopt the change will affect its success or failure. Plan to hold meetings by stakeholder groups with about five to ten stakeholders, including at least one manager, three to five clinic staff members, and two to three patients.

2. Hold group meetings of stakeholders to identify the considerations they have, both positive and negative, about the change you propose.

Have one meeting for each stakeholder group, with about five to nine participants in each group. Have a flipchart or whiteboard on hand. Use Nominal Group Technique to elicit the group's values, using these steps:

a. Start by briefly explaining the change you want to implement. Then explain that you want to know what considerations come into play—what pros and cons come to mind—when participants think about making that change (e.g., the time it will take, its value to patients, etc.)

b. Ask participants to spend two minutes writing down their ideas in phrases.

c. Ask participants to go around the table saying one idea they wrote down. Record each idea on the flipchart or whiteboard. Record the ideas verbatim, without paraphrasing them, though you can ask for clarification if needed.

d. After everyone's ideas are recorded, briefly discuss each idea to be sure the ideas are clear to participants.

e. Give participants time to silently and independently choose the most important three considerations from those on the flipchart or whiteboard. Then have participants go to the flipchart or whiteboard and put a mark next to the considerations they thought to be the three most important. Tally the marks next to each item to determine the most important ideas. The number of most important ideas will vary depending on how many different considerations participants listed and how much agreement they had, as a group, about the most important ones.

3. Weigh stakeholders' considerations to arrive at the value they attach to the change.

For each stakeholder group, select from three to six of the most important considerations. Label each as a *Gain* (or *Pro*) or *Loss* (*Con*). Then give each of these considerations a weight proportional to its rating within the set of ideas. For example, if you have a group of five most important considerations, you have 5+4+3+2+1=15 points available to distribute among the ideas. The consideration ranked most important receives a weight of 5 out of 15, followed by 4 out of 15 for the next most important consideration, and so on, and the weight is positive or negative depending on whether it is a gain or a loss. Finally, add up the weight for all the gains and for all the losses and multiply each weight by the total points available to distribute among the ideas (15). *Value* is determined by subtracting perceived losses from perceived gains. If the gains outweigh the losses, stakeholders give a positive value to the change. If gains and losses are the same, stakeholders' value is neutral. If losses outweigh gains, stakeholders' value is negative. Here's an example of the considerations clinic staff had when they considered adopting a mobile health system to help treat their patients with addiction.

Gains and losses by importance	Assigned weight	Total value
1. Loss: additional time required to learn and use a new system	-5	11 of 15 available points are negative.<br><br>4 of 15 available points are positive.<br><br>Net value is negative: -7.
2. Loss: disruption of current workflows, including integration with the electronic health record (EHR)	-4	
3. Gain: improved quality of patient care	+3	
4. Loss: uncertainty about long-term sustainability	-2	
5. Gain: potential to automate clinical functions currently done manually	+1	

4. Use the values to identify actions to improve the likelihood of adoption.

The purpose of identifying the values of different stakeholders is not just to have the information—it's to *use* it to guide action. Use any stakeholder group's neutral or negative value as a sign to modify the change you advocate and/or how you will suggest implementing it.

Examples

- Clinic staff express concern about the additional time required to implement a change in practice. In response, a manager or managers could sit down with two or three clinicians to identify the work required and then set aside a specific time—an hour every Friday morning, for example—to do the work.
- A payer thinks the potential change will end up costing rather than saving money. In response, the proposed change could be adopted in a small pilot test and costs carefully tracked. If costs turn out to be too great, adjustments could be made to the change or, if savings aren't possible, the change could be abandoned.
- Patients think a change will take more time than they want to spend on it. In response, clinicians could ask a few patients to identify exactly which aspects of the change will take too much time and see if these aspects could be changed or dropped.
